# Draft genome sequence of *Hericium erinaceus* strain BCRC 35669

**DOI:** 10.1128/mra.00536-26

**Published:** 2026-10-07

**Authors:** Shiu-Nan Chen, Ya-Chih Chang, Min-Feng Yang, Ching-Sheng Chang, Sherwin Chen

**Affiliations:** 1 College of Life Science, National Taiwan University 124716, Taipei, Taiwan; 2 Department of Research and Development, Super Beta Glucan Inc., Irvine, California, USA; University of California Riverside, Riverside, California, USA

**Keywords:** whole-genome sequencing, *Hericium erinaceus*, food microbiology

## Abstract

The study reports a high-quality draft genome sequence of *Hericium erinaceus* strain BCRC 35669 generated using hybrid Illumina and Oxford Nanopore sequencing. The assembly comprises 222 contigs with 94.3% completeness and did not reveal secondary metabolite pathways associated with known mycotoxins or virulence-related genes of concern.

## ANNOUNCEMENT

*Hericium erinaceus* (Lion’s Mane) is an edible and therapeutically used fungus in the family Hericiaceae with reported neuroprotective, immunomodulatory, and gastrointestinal health-promoting properties (1–3). Strain BCRC 35669 was isolated in 1998 from a fruiting body collected on the trunk of an angiosperm at Piluhsi (Provincial Highway No. 8, kilometer 103; altitude 2,100 m) in Nantou County, Taiwan. Here, we present the draft genome assembly of *H. erinaceus* strain BCRC 35669 obtained from the Bioresource Collection and Research Center (BCRC), Taiwan.

The strain was taxonomically identified by multilocus sequencing using a concatenated data set of RNA polymerase II second subunit (RPB2), internal transcribed spacer (ITS), and translation elongation factor 1-alpha (TEF1-α) genes, together with alignment-free genome distance estimation, showing highest similarity to *H. erinaceus* reference sequences. Mycelia were harvested after 28 days of growth on yeast malt (YM) agar at 25°C. Genomic DNA was extracted from lyophilized cells using the FavorPrep Fungi/Yeast Genomic DNA Extraction Kit (FAVORGEN Biotech Corp., Taiwan). Whole-genome sequencing was performed using a GridION platform (Oxford Nanopore Technologies) and a NovaSeq X platform (Illumina) with 2 × 150 bp paired-end reads. For ONT sequencing, high-molecular weight DNA was used for library preparation with the ligation-based Ligation Sequencing Kit V14 (SQK-LSK114; Oxford Nanopore Technologies, UK). Library construction followed the manufacturer’s protocol using the NEBNext Companion Module v2 for Oxford Nanopore Technologies Ligation Sequencing Kit (E7672; New England Biolabs, USA). DNA was not intentionally sheared prior to library preparation. Illumina libraries were prepared using the Illumina DNA Prep Kit (Illumina, USA) according to the manufacturer’s instructions.

Short reads were trimmed using fastp v0.23.4 (4). Long reads were basecalled using Dorado v0.8.3 on a FLO-MIN114 R10.4.1 flow cell (5), followed by adapter trimming with Porechop v0.2.4 (6), filtering with Filtlong v0.2.1 (7), and subsampling to 100× coverage using rasusa v0.3.0 (8). Read quality was assessed using FastQC v0.12.1 (9) and NanoPlot v1.41.6 (10). Long reads were assembled using wtdbg2 v2.5 (11) and polished with short reads using Hapo-G v1.3.8 (12). Potential contamination was assessed using MMseqs2 v2.0.8 (13). No additional read-length or quality-score thresholds were specified during trimming, and genome assembly was performed without custom parameter optimization.

The assembly resulted in 222 contigs with a total length of 45,596,692 bp, an N50 value of 907,297 bp, and a guanine-cytosine (GC) content of 52.3% (Table 1). Genome annotation predicted 11,914 genes, including 11,667 protein-coding sequences, 247 tRNAs, and no rRNAs.

**TABLE 1:** Summary of genome assembly and annotation of *H. erinaceus* BCRC 35669

Parameter	Value
Illumina sequencing	
Total number of reads	6,545,416 (2 × 150 bp paired reads)
Total bases (bp)	1,931,756,926 (965,557,789 + 966,199,137)
Coverage	42×
Nanopore sequencing	
Total number of reads	2,806,500
Total bases (bp)	14,984,027,840
ONT read N50 (bp)	13,879
Coverage	87×
Genome assembly	
Total length (bp)	45,596,692
Number of contigs	222
N50 (bp)	907,297
GC content (%)	52.3
Complete BUSCOs (%)	94.3%

Gene prediction and functional annotation were performed using Funannotate v1.8.9 (14) and Liftoff v1.6.3 (15). The genome was screened for secondary metabolite biosynthetic gene clusters (BGCs) using antiSMASH v7.0.0 (16) with the MiBIG v4.0 (17) database and for virulence- and toxicity-related genes using UniProtKB (18) and the Database of Fungal Virulence Factors (DFVF) (19). Twenty BGCs were identified, with none showing significant similarity to known mycotoxin- or virulence-associated clusters and no homologs of known virulence factors or toxin-encoding genes being detected. This genotypic characterization, concordant with the strain’s documented phenotypic profile, supports the absence of known virulence- or toxin-associated features in *H. erinaceus* BCRC 35669.

## Data Availability

This Whole-Genome Shotgun project has been deposited in DDBJ/ENA/GenBank under accession number JBUEFX000000000. The version described in this paper corresponds to the updated assembly available under accession number JBUEFX010000000. The BioProject accession number is PRJNA1379243, and the BioSample accession number is SAMN53867182. The raw sequencing reads have been deposited in the Sequence Read Archive (SRA) under accession numbers SRR37879207 and SRR37879208.
